# Treatment-emergent depression and anxiety between peginterferon alpha-2a versus alpha-2b plus ribavirin for chronic hepatitis C

**DOI:** 10.1186/s12888-016-1135-8

**Published:** 2016-11-25

**Authors:** Liang-Jen Wang, Shuo-Wei Chen, Chih-Ken Chen, Cho-Li Yen, Jia-Jang Chang, Tsung-Shih Lee, Ching-Jung Liu, Li-Wei Chen, Rong-Nan Chien

**Affiliations:** 1 Department of Child and Adolescent Psychiatry, Kaohsiung Chang Gung Memorial Hospital and Chang Gung University College of Medicine, Kaohsiung, Taiwan; 2 Liver Research Unit, Chang Gung Memorial Hospital, Chang Gung University College of Medicine, 222 Mai-Chin Road, Keelung, Taiwan; 3 Department of Psychiatry, Chang Gung Memorial Hospital, Keelung, Taiwan; 4 Chang Gung University College of Medicine, Taoyuan, Taiwan

**Keywords:** Anxiety, Depression, Interferon, Ribavirin, Hepatitis C

## Abstract

**Background:**

This study investigates differences in depression and anxiety between patients with chronic hepatitis C who are treated with peginterferon alpha-2a (PegIFN-α-2a) plus ribavirin and those who are treated with peginterferon alpha-2b (PegIFN-α-2b) plus ribavirin.

**Methods:**

In this 24 week, non-randomized, observational, prospective study, 55 patients with chronic hepatitis C were treated with PegIFN-α-2a plus ribavirin (Group 1), and 26 patients were treated with PegIFN-α-2b plus ribavirin (Group 2). All patients underwent assessment using the Hospital Anxiety and Depression Scale (HADS) at the baseline and at weeks 4, 12 and 24. Patients with depression scores (HADS-D) ≥ 8 and anxiety scores (HADS-A) ≥ 8 were defined as having depression and anxiety, respectively. The factors that were associated with depression and anxiety during the 24 week antiviral treatment were determined.

**Results:**

During the 24 week antiviral treatment, the proportion of patients with depression significantly increased over time in both groups (Group 1: *p* = 0.048; Group 2: *p* = 0.044). The proportion of patients with anxiety did not significantly change during the follow-up period in either group. Incidences of depression or anxiety did not differ significantly between Group 1 and Group 2. A history of alcohol use disorder was an independent predictor of depression at week 12 (*p* < 0.001) and week 24 (*p* < 0.001), and a poor virological response to treatment was associated with depression at week 24 (*p* = 0.029). Patients who had more physical comorbidities were more likely to suffer from anxiety at week 12 (*p* = 0.038).

**Conclusions:**

This study did not identify significant differences in depression or anxiety between in patients with chronic hepatitis C who underwent a 24 week antiviral treatment regimen with PegIFN-α-2a plus ribavirin and those who underwent a regiment with PegIFN-α-2b plus ribavirin. Future research with larger samples and a randomized, controlled design are required to verify the findings in this study.

**Trial registration:**

This clinical study has been registered at ClinicalTrials.gov. (Trial registration: NCT02943330).

## Background

Chronic infection with hepatitis C virus (HCV) is a serious health problem worldwide. Chronic hepatitis C increases the risk of morbidity and mortality from sequelae such as liver cirrhosis and hepatocellular carcinoma [1]. The purpose of treatment is the prevention of complications of chronic HCV infection, which is principally achieved by eradicating the infection [2]. A combination of weekly subcutaneous injections of long-acting peginterferon alfa (PegIFN-α) and oral ribavirin greatly improves the efficacy of antiviral treatment and represents the current standard of care for patients with chronic hepatitis C [2]. Depression is one of the most common neuropsychiatric complications during antiviral treatment for chronic hepatitis C [3–7]. A meta-analysis has found that around 25% of chronic hepatitis C patients who receive interferon and ribavirin treatment will develop a treatment-induced major depressive episode [6]. Although interferon exposure is clearly associated with an increased incidence of depression, the relationships of the frequency and magnitude of depression with the specific type of interferon used have seldom been evaluated [5].

The two available licensed products of PegIFN-α are peginterferon alfa-2a (PegIFN-α-2a) and peginterferon alfa-2b (PegIFN-α-2b), which differ in their pharmacokinetics and chemical properties; both are cost-effective and recommended for use in combination with ribavirin for treating chronic hepatitis C [8]. Several researchers have found that, when combined with ribavirin (RBV), PegIFN-α-2a yields a higher virological response rate than does PegIFN-α-2b, [9, 10]. However, one review article indicated that PegIFN-α-2a and PegIFN-α-2b exhibit similar degrees of effectiveness and tolerability, with few clinical differences [11], while investigations have suggested similar sustained virological response (SVR) rates and frequencies of adverse events upon treatment with PegIFN-α-2a to those with -2b [11–13]. Compared to those treated with PegIFN-α-2a plus RBV, some clinical trials have indicated that patients treated with PegIFN-α-2b plus RBV have similar incidence rates of depression [14, 15], whereas one revealed that patients administrated with PegIFN-α-2b plus RBV have higher probability of depression [16]. To date, no conclusive evidence has established that one PegIFN-α is superior to the other, or that one type of PegIFN-α is more likely to induce depression than the other when combined with RBV in the treatment of chronic hepatitis C.

This study investigates the differences in depression and anxiety between patients with hepatitis C who receive PegIFN-α-2a plus RBV and those who receive PegIFN-α-2b plus RBV. The factors that are associated with depression and anxiety are examined.

## Methods

### Participants and procedures

The study was conducted according to the ethical guidelines of the Good Clinical Practice and was approved by the Institutional Review Board of Chang Gung Memorial Hospital. Each patient who was enrolled in this study gave written informed consent. This clinical study has been retrospectively registered at ClinicalTrials.gov. (Trial registration: NCT02943330).

In this prospective study, 81 patients who were diagnosed with hepatitis C (aged ≥18 years) and received PegIFN plus RBV combination therapy were enrolled at Keelung Chang Gung Memorial Hospital (the largest general hospital in North-East Taiwan). All 81 patients received a 24-week combination treatment with a weekly subcutaneous PegIFN injection plus daily oral RBV. The treatment regimen is consistent with the reimbursement criteria of the National Health Insurance in Taiwan. The prescribed PegIFN regimen was either 180 μg of PegIFN-α-2a, or 1.5 μg/kg (weight-based) PegIFN-α-2b. The treatment regimens were allocated by the physicians’ decision, based on patients’ body weight and clinical manifestations. In cases of genotype 1 HCV infection, the oral RBV dose was 1000 mg daily for patients with body weight (BW) <75 kg or 1200 mg for those with BW ≥75 kg. If genotype non-1 HCV-infection, the RBV dose was 800 mg daily. After the end of the treatment, all patients were followed up for 24 weeks.

In the treatment period, patients made weekly outpatient visits for the first four weeks, biweekly visits between the fifth and 12th weeks, and monthly visits for the last 12 weeks. During the 24 week follow-up period, patients attended the clinic monthly or bimonthly. Hepatic biochemical tests were performed on each visit. HCV RNA levels were measured at the beginning of treatment, the end of treatment and 24 weeks after treatment. All biochemical and virological tests were carried out in the clinical laboratories of Chang Gung Memorial Hospital at Keelung. Adverse effects of antiviral treatment were recorded at each visit. Of all enrolled patients, 55 received combination treatment with PegIFN-α-2a plus RBV (Group 1), and 26 received combination treatment with PegIFN-α-2b plus RBV (Group 2).

This study was a 24 week, observational, non-randomized prospective study. At the baseline (pretreatment), patients’ mood-related symptoms were assessed. Physicians reassessed patients in both groups at the outpatient department weekly. The second, third and fourth study assessments were conducted at weeks 4, 12 and 24 after the antiviral treatment had begun. The assessment of mood-related symptoms at the baseline was conducted at each time-point in the study assessments.

### HCV genotyping and treatment response

Anti-HCV tests were carried out using a third-generation enzyme immunoassay kit (Abbott Laboratories, Berkshire, UK). Quantity of serum HCV RNA was determined using a real-time polymerase chain reaction (PCR) assay (Roche Molecular Systems, Inc., Branchburg, NJ, USA), with a detection limit of 15 IU/mL. HCV genotype was identified using a linear probe assay (Bayer Corporation, NY, USA). The HCV RNA load was examined before treatment, 4 weeks during treatment, end of treatment and 24 weeks after treatment. The treatment efficacy was assessed after 24 weeks of combined PegIFN plus RBV therapy. Patients who were HCV RNA-negative at week 24 post-treatment follow-up were regarded as sustained virological response (SVR).

### Evaluation of psychological well-being

Charts were reviewed to collect information about patients’ alcohol use disorder and comorbid physical illness. The mood-related symptoms of all patients were assessed on the Hospital Anxiety and Depression Scale (HADS) [17]. The HADS is frequently used in hospital practice and primary care, as well as for the general population. Of the 14 items, seven items are used to evaluate anxiety and the rest seven items are used to assess depression. There are four possible responses (scored 0–3) for each item; the anxiety and depression subscales are counted as independent measures. A patient with an anxiety score (HADS-A) ≥ 8 is deemed to have an anxiety disorder (sensitivity, 0.89; specificity, 0.75) and a patient with a depression score (HADS-D) ≥ 8 is deemed to have a depression disorder (sensitivity, 0.80; specificity, 0.88) [18]. The validation and cut-off score of Chinese version of the HADS have been reported in previous studies [19, 20].

### Statistical analysis

Data analysis was performed using the Statistical Package for the Social Sciences for Windows (version 16.0; SPSS, Inc., Chicago, IL, USA). Continuous variables and categorical variables are presented as either mean ± standard deviation (SD) and as frequency, respectively. Categorical variables between Group 1 and Group 2 were examined using either the chi-square (*χ* ^*2*^) test or Fisher’s exact test, depending on case numbers. An independent *t*-test or Mann–Whitney *U*-test was applied to compare continuous variables between groups. All statistical tests were two-tailed, and the significant levels were set as *p* < 0.05.

The depression and anxiety states of patients were determined using cut-off points of HADS-D (≥8) and HADS-A (≥8), respectively. The chi-square tests or Fisher’s exact tests were carried out to determine whether the proportion of patients with depression or anxiety significantly changed over time. The trends of HADS scores (continuous variable) during the 24 weeks of treatment were also analyzed. Generalised estimating equations (GEE) were used to perform a *post hoc* analysis of the difference in the depression and anxiety scores between pairs of time-points and between treatment groups, followed by a *post hoc* analysis using least significant difference (LSD) test. To identify the factors that were associated with depression and anxiety during combination treatment, GEE, the maximum likelihood estimation method with an auto-regression covariance matrix, were used. Degrees of depression and anxiety were set as dependent variables. Demographic variables, physical conditions and treatment response were set as independent variables. Since the attrition rate was high by week 24, completer analyses were conducted for patients who remained in the study at week 12 and at week 24, respectively.

## Results

This study involved 55 patients with hepatitis C infection who underwent combination treatment with PegIFN-α-2a plus RBV (Group 1) and 26 patients who underwent combination treatment with PegIFN-α-2b plus ribavirin (Group 2). Table 1 summarizes the baseline demographic characteristics and mood statuses of the two patient groups. At the beginning of combination therapy 14 (25.5%) of patients in Group 1 and 3 (11.5%) of patients in Group 2 have been classified as depression cases (HADS-D ≥ 8), and 21 (38.2%) of patients in Group 1 and 6 (23.1%) of patients in Group 2 have been classified as anxiety cases (HADS-A ≥ 8). The mean dose of RBV that was co-administrated for hepatitis C treatment was higher for Group 1 (1048.2 mg/day) than for Group 2 (958.3 mg/day). There was no significant difference in other variables.

**Table 1:** Baseline characteristics of patients with hepatitis C virus (HCV) infection who were treated with PegIFN-α-2a plus ribavirin (Group 1) and those who were treated with PegIFN-α-2b plus ribavirin (Group 2)

Characteristics	Group 1 (*N* = 55)	Group 2 (*N* = 26)	Statistic Value	*P*-value
Sex (*n*, %)			0.045 ^b^	0.831
Female	31 (56.4)	14 (53.8)		
Male	24 (43.6)	12 (46.2)		
Age at recruitment (years)	52.8 ± 12.2	55.0 ± 11.5	0.747 ^c^	0.457
Body weight (kg)	65.7 ± 12.1	63.8 ± 12.8	0.644 ^c^	0.521
Alcohol use disorder (*n*, %)	7 (12.7)	1 (3.8)	1.564 ^d^	0.426
Number of physical illness	2.8 ± 1.8	2.4 ± 1.2	1.287 ^c^	0.203
α-fetoprotein level (ng/mL)	10.6 ± 15.8	7.6 ± 5.9	0.903 ^c^	0.369
Genotype of HCV ^a^			0.104 ^b^	0.747
1	27 (50)	14 (53.8)		
Non-1	27 (50)	12 (46.2)		
PegIFN-α-2a dose (mcg/week)	175.1 ± 14.2	-	N/A	N/A
PegIFN-α-2b dose (mcg/week)	-	94.8 ± 12.4	N/A	N/A
Ribavirin dose (mg/day)	1048.2 ± 164.5	958.3 ± 131.6	2.357 ^c^	0.021^*^
HADS-Depression score	5.0 ± 3.8	4.0 ± 3.1	1.008 ^e^	0.314
Depressive state (*n*, %)	14 (25.5)	3 (11.5)	2.062 ^d^	0.242
HADS-Anxiety score	6.6 ± 4.1	6.7 ± 4.7	0.193 ^e^	0.847
Anxiety state (*n*, %)	21 (38.2)	6 (23.1)	1.813 ^b^	0.214

*Note*: Data is expressed by *n* (%) or mean ± SD

*PegIFN-α-2a*: Pegylated interferon-α-2a, *PegIFN-α-2b*: Peginterferon-α-2b, *HADS*: the Hospital Anxiety and Depression Scale, *N/A*: non-applicable

*p < 0.05

^a^HCV genotyping of one patient in Group 1 was undetermined

^b^Chi-square test (degrees of freedom = 1)

^c^Fisher’s exact test

^d^t-test (degrees of freedom = 79)

^e^Mann–Whitney *U*-test

Of the 55 patients in Group 1, 52 (94.5%), 51 (92.7%) and 36 (65.5%) remained in the study until weeks 4, 12 and 24, respectively. Of the 26 patients in Group 2, 22 (84.6%), 19 (73.1%) and 15 (57.7%) remained in the study until weeks 4, 12 and 24, respectively. After six months of treatment, 22 (61.1%) patients in Group 1 and nine (60%) patients in Group 2, respectively, were SVR (HCV RNA-negative). The attrition rates (*χ* ^*2*^ = 0.46, *p* = 0.499) and treatment response (SVR) rates (*χ* ^*2*^ = 0.10, *p* = 0.753) did not differ significantly between the two groups at the endpoint.

**Fig. 1:**
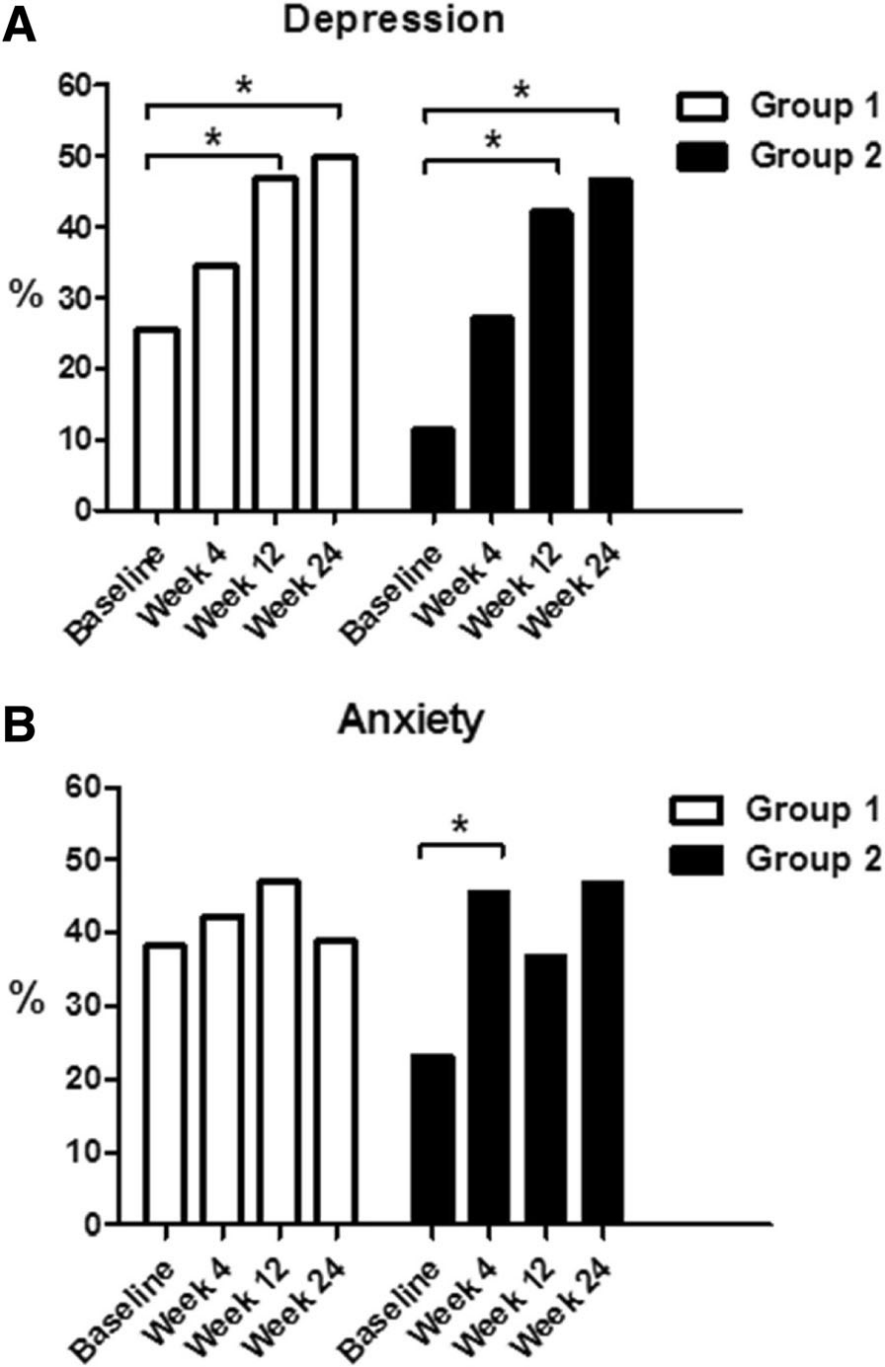
The proportion of depression (**a**) and anxiety (**b**) among patients during a combination treatment with interferon and ribavirin at the baseline, week 4, 12 and 24. *Note:* Group 1, patients who received antiviral treatment with PegIFN-α-2a plus ribavirin; Group 2, patients who received antiviral treatment with PegIFN-α-2b plus ribavirin. There were no significant differences in the proportion of depression or anxiety between the two groups at any time-point. ^*^ *p* < 0.05 in comparison to the proportion at baseline

Figure 1 presents the proportion of patients in the two groups with depression and anxiety during the 24 week follow-up. We found the proportion of depression cases significantly increased in both groups (Group 1: *χ* ^*2*^ = 7.89, *p* = 0.048; Group 2: *χ* ^*2*^ = 7.91, *p* = 0.044). The *post hoc* test revealed that the trends of depression in both groups (week 12 > baseline; week 24 > baseline) were similar. The proportion of anxiety cases did not significantly change throughout the overall follow-up period in either group (Group 1: *χ* ^*2*^ = 1.01, *p* = 0.798; Group 2: *χ* ^*2*^ = 3.45, *p* = 0.321). The *post hoc* test revealed that anxiety in Group 2 was more prevalent at week 12 than at the baseline (*β* = 2.50, *p* = 0.018). No significant difference was observed in any other pairwise comparisons between time-points or the two groups.

The HADS-D scores in both groups significantly increased over time (Group 1: Wald *χ* ^*2*^ = 12.78, *p* = 0.005; Group 2: Wald *χ* ^*2*^ = 9.88, *p* = 0.020). The *post hoc* test revealed that the HADS-D scores in Group 1 significantly increased after week 4 (*p* < 0.05) while those in Group 2 significantly increased after week 12 (*p* < 0.05). The HADS-A scores did not significantly increase over time in either group (Group 1: Wald *χ* ^*2*^ = 3.04, *p* = 0.385; Group 2: Wald *χ* ^*2*^ = 1.76, *p* = 0.623). A *post hoc* test also revealed that HADS-A scores did not differ significantly between time-points or groups.

Table 2 lists the factors that were associated with patients’ depression at week 12 and at 24 week of combination therapy. Alcohol use disorder was consistently associated with depression at week 12 (*β* = 6.59, *p* < 0.001) and at week 24 (*β* = 5.96, *p* < 0.001). Poor response to combination therapy (HCV RNA positive) was significantly associated with patients’ depression at week 24 (*β* = 2.43, *p* = 0.029).

**Table 2:** Factors associated with depression during an antiviral treatment for hepatitis C virus (HCV) infection at week 12 and at week 24

Characteristics	Week 12			Week 24		
	*β* (95% CI)	Wald *χ* ^2^	*p*-value	*β* (95% CI)	Wald *χ* ^2^	*p*-value
Treatment group (Group 1 vs. Group 2)	1.33 (0.59–3.00)	0.475	0.490	1.09 (0.51–2.31)	0.046	0.830
Sex (female vs. male)	1.70 (0.69–4.18)	1.335	0.248	1.55 (0.70–3.43)	1.142	0.285
Age at recruitment (year)	0.98 (0.95–1.02)	1.012	0.315	0.99 (0.96–1.02)	0.887	0.346
Body weight (kg)	0.97 (0.94–1.01)	1.956	0.162	0.98 (0.94–1.01)	1.653	0.199
Alcohol use disorder	6.59 (2.31–18.74)	12.474	<0.001^**^	5.96 (2.21–16.09)	12.424	<0.001^**^
Number of physical comorbidities	1.25 (0.99–1.59)	3.365	0.067	1.23 (0.97–1.57)	2.901	0.089
α-fetoprotein level (ng/mL)	1.00 (0.99–1.02)	0.037	0.847	0.98 (0.96–1.01)	1.589	0.208
Genotype of HCV (non- 1 vs. 1)	1.31 (0.59–2.92)	0.446	0.504	1.57 (0.72–3.44)	1.275	0.259
Ribavirin daily dose (g/day)	1.49 (0.40–5.59)	0.343	0.558	2.23 (0.87–5.67)	2.802	0.094
Poor treatment response (HCV RNA positive)	1.70 (0.76–3.82)	1.658	0.198	2.43 (1.09–5.41)	4.748	0.029^*^

*Note*: Group 1, patients who were treated with PegIFN-α-2a plus ribavirin; Group 2, patients who were treated with PegIFN-α-2b plus ribavirin; Depression, HADS depression score ≥ 8; 95% CI, 95% confidence interval

^*^ *p* < 0.05, ^**^ *p* < 0.001

Table 3 shows factors that are associated with patients’ anxiety at week 12 and at week 24 of combination treatment. Patients’ physical comorbidities were positively correlated with their anxiety status at week 12 (*β* = 1.28, *p* = 0.038). Nonetheless, no variable was significantly associated with anxiety at week 24.

**Table 3:** Factors associated with anxiety during an antiviral treatment for hepatitis C virus (HCV) infection at week 12 and at week 24

Characteristics	Week 12			Week 24		
	*β* (95% CI)	Wald *χ* ^2^	*p*-value	*β* (95% CI)	Wald *χ* ^2^	*p*-value
Treatment group (Group 1 vs. Group 2)	1.07 (0.45–2.56)	0.021	0.884	0.88 (0.38–2.02)	0.088	0.767
Sex (female vs. male)	0.89 (0.34–2.35)	0.055	0.815	0.90 (0.38–2.14)	0.059	0.808
Age at recruitment (year)	0.97 (0.94–1.01)	1.911	0.167	0.98 (0.94–1.01)	1.899	0.168
Body weight (kg)	0.99 (0.95–1.02)	0.624	0.430	0.99 (0.95–1.03)	0.326	0.568
Alcohol use disorder	2.07 (0.58–7.42)	1.235	0.266	1.48 (0.46–4.78)	0.430	0.512
Number of physical comorbidities	1.28 (1.01–1.62)	4.303	0.038*	1.21 (0.96–1.53)	2.581	0.108
α-fetoprotein level (ng/mL)	1.00 (0.99–1.02)	0.123	0.726	0.98 (0.94–1.01)	2.403	0.121
Genotype of HCV (non- 1 vs. 1)	0.66 (0.27–1.63)	0.801	0.371	0.70 (0.28–1.72)	0.615	0.433
Ribavirin daily dose (g/day)	1.20 (0.24–6.00)	0.052	0.820	1.95 (0.67–5.64)	1.505	0.220
Poor treatment response (HCV RNA positive)	1.45 (0.60–3.50)	0.669	0.414	1.53 (0.61–3.82)	0.832	0.362

*Note:* Group 1, patients who were treated with PegIFN-α-2a plus ribavirin; Group 2, patients who were treated with PegIFN-α-2b plus ribavirin; Anxiety, HADS anxiety score ≥ 8; 95% CI, 95% confidence interval

^*^ *p* < 0.05

## Discussion

During 24 weeks of antiviral treatment, the proportion of depression cases significantly increased in both groups over time. Significant differences in depression or anxiety levels were not observed between patients with chronic hepatitis C who underwent 24 week antiviral treatment with PegIFN-α-2a plus RBV and those who underwent such treatment with PegIFN-α-2b plus RBV. A history of alcohol use disorder was an independent predictor of depression at week 12 and week 24 of combined PegIFN plus RBV treatment and a poor virological response to treatment was associated with depression at week 24. Additionally, patients who had more physical comorbidities were more likely to exhibit anxiety at week 12.

During the 24 weeks of antiviral treatment, the proportion of patients with depression significantly increased over time, in line with previous reviews and meta-analysis [5, 6]. Newly developed depression was observed in 33 patients during antiviral treatment. Nevertheless, we found that 8 patients whose HADS depression scores were above the cut-off at baseline had mood improved during treatment. Although the relative efficacies of different forms of PegIFN-α are currently debated, the data herein reveal that neither the virological response rate nor trend in mental health status, differed between patients who were treated with PegIFN-α-2a (Group 1) and those who were treatment with PegIFN-α-2b (Group 2). Notably, this study used non-randomized and open-label methods. At the baseline, patients in Group 2 seemed to have a lower anxiety level, although not significantly, than those in Group 1. Therefore the possibility of selection bias cannot be excluded. Randomized trials have revealed that patients who are given PegIFN-α-2a plus RBV and those who are given PegIFN-α-2b plus RBV exhibit similar incidences of depression [14, 15]. However, one trial demonstrated that patients who were administered PegIFN-α-2b plus RBV were more likely to develop depression than counterparts who were administered PegIFN-α-2a plus RBV [16]. Therefore, a future study which conducted in multi-centre, with randomized controlled design and adequate stratification for controlling the potential confounding variables (e.g. mood status at baseline, HCV genotype, prior psychiatric history) is warranted to determine whether PegIFN-α variants have differently alter mental health.

Studies have established that relatively many patients with chronic hepatitis C have alcohol use disorder [21]. Alcohol use disorder is associated with increased use of illicit substances and impulsive behaviors [22] that may increase the likelihood of HCV acquisition [23]. Furthermore, alcohol use can worsen hepatic problems and can lead to a negative effect on adherence to a treatment regimen and responses to antiviral therapy [23]. This study revealed that a history of alcohol use disorder predicted depression during 24 weeks of combination treatment with PegIFN and ribavirin for chronic hepatitis C. Alcohol abuse can worsen hepatic disease and reduce adherence to a treatment regimen. Therefore, the identification and treatment of alcohol abuse in this population is very important.

The results of this study indicated that a poor virological response to combination treatment with interferon and RBV for chronic hepatitis C is associated with depression at week 24. The possibility of a causal relationship between the response to antiviral treatment and depression is intriguing. While treatment with interferon-α is known potentially to cause neuropsychiatric side effects, however, the neuropsychiatric symptoms is reduced in HCV patients achieving an SVR [24]. HCV infection is regarded as a systemic disease because liver disease is accompanied by effects on other organs and tissues. The brain is a favorable site for HCV replication, and the virus may be directly neurotoxic. Other mechanisms that have been proposed to explain the pathophysiology of neuropsychiatric disorders in cases of chronic HCV infection, such as derangement in neurotransmitter circuits, inflammation and autoimmune dysregulation and alterations of metabolic pathways of infected cells. Improvement of neuropsychiatric symptoms in patients achieving SVR following interferon treatment has been noticed [25–28]. Some other factors may favor depression and the poor outcome of antiviral treatment. For example, depression is associated with poor adherence or early discontinuation of antiviral treatment in cases of chronic hepatitis C [15]. Additionally, patient satisfaction owing to awareness of being cured of hepatitis C may bias the results of this study. Therefore, care should be taken in interpreting the association between depression and a poor virological response to treatment.

The results herein also demonstrate that the number of co-morbid physical illnesses was significantly correlated with anxiety at week 12. Previous studies have found that 24% to 36% of patients who undergo interferon-α treatment for HCV infection suffer from anxiety disorders [29, 30]. However, no specific risk factor for anxiety disorders has been identified [29]. We propose the possible explanation is patients with many comorbidities related physical illnesses, not only having a poorer physical condition, but also suffering from a greater psychological stress. Patients with multiple physical comorbidities may be vulnerable to the adverse effects of PegIFN-α, which are linked to a high probability of anxiety.

Several limitations of this study should be concerned. First, the sample size was small - especially in Group 2, and a smaller sample provides less statistical power to detect potential differences in mental health between treatment groups, and limits the ability to assess change in anxiety score. Second, this study was a naturalistic observational study, rather than a randomized controlled trial, hence the results may be influenced by selection bias. Additionally, the attrition of patients underwent antiviral treatment could be related to lack of effect, adverse effect, poor adherence to the prescription or simply loss of follow up. The sample sizes in the two treatment groups were small and unequal resulting certain attrition rates lead to inability for a subgroup analysis. Third, various important factors that may have affected patients’ mental health were not identified, such as compliance with the PegIFN-α and RBV regimen, the presence of infectious diseases, and the taking of other drugs (such as antidepressants). It is uncertain whether such factors influenced the results of this study. Finally, the assessments that were made using the HADS covered a period of one week, and do not by themselves constitute a psychiatric diagnosis of a major depressive disorder, which requires a depressed mood or anhedonia with other critical associated symptoms that are present for at least two weeks. Cut-off points of the HADS were used to establish depression or anxiety without a formal confirmation of the diagnosis based on DSM-IV-TR criteria through a structured interview, although higher HADS scores commonly correlate with a diagnosis of a major depressive disorder or anxiety disorders [18].

## Conclusion

This investigation did not reveal significant differences in depression or anxiety levels of patients with chronic hepatitis C on a 24 week antiviral treatment regimen with PegIFN-α-2a plus RBV and those on such a corresponding regiment with PegIFN-α-2b plus RBV. These two regimens worsened HCV patients’ depression and anxiety. Future research with larger samples and a randomized, controlled design are warranted to verify the findings of this study.

## Abbreviations

GEE: Generalized estimating equations
HADS: Hospital Anxiety and Depression Scale
HCV: Hepatitis C virus
PegIFN-α: Peginterferon alfa
RBV: Ribavirin
SVR: Sustained virological response

## References

[1] Alter MJ (2007). Epidemiology of hepatitis C virus infection. World J Gastroenterol.

[2] Strader DB, Wright T, Thomas DL, Seeff LB (2004). Diagnosis, management, and treatment of hepatitis C. Hepatology.

[3] Dieperink E, Willenbring M, Ho SB (2000). Neuropsychiatric symptoms associated with hepatitis C and interferon alpha: A review. Am J Psychiatry.

[4] Horikawa N, Yamazaki T, Izumi N, Uchihara M (2003). Incidence and clinical course of major depression in patients with chronic hepatitis type C undergoing interferon-alpha therapy: a prospective study. Gen Hosp Psychiatry.

[5] Asnis GM, De La Garza R (2006). Interferon-induced depression in chronic hepatitis C: a review of its prevalence, risk factors, biology, and treatment approaches. J Clin Gastroenterol.

[6] Udina M, Castellvi P, Moreno-Espana J, Navines R, Valdes M, Forns X (2012). Interferon-induced depression in chronic hepatitis C: a systematic review and meta-analysis. J Clin Psychiatry.

[7] Lotrich FE, Rabinovitz M, Gironda P, Pollock BG (2007). Depression following pegylated interferon-alpha: characteristics and vulnerability. J Psychosom Res.

[8] Sullivan SD, Jensen DM, Bernstein DE, Hassanein TI, Foster GR, Lee SS (2004). Cost-effectiveness of combination peginterferon alpha-2a and ribavirin compared with interferon alpha-2b and ribavirin in patients with chronic hepatitis C. Am J Gastroenterol.

[9] Yang Z, Zhuang L, Yang L, Chen X (2013). Efficacy and Tolerability of Peginterferon alpha -2a and Peginterferon alpha -2b, Both plus Ribavirin, for Chronic Hepatitis C: A Meta-Analysis of Randomized Controlled Trials. Gastroenterol Res Pract.

[10] Hauser G, Awad T, Thorlund K, Stimac D, Mabrouk M, Gluud C (2014). Peginterferon alpha-2a versus peginterferon alpha-2b for chronic hepatitis C. Cochrane Database Syst Rev.

[11] Foster GR (2010). Pegylated interferons for the treatment of chronic hepatitis C: pharmacological and clinical differences between peginterferon-alpha-2a and peginterferon-alpha-2b. Drugs.

[12] Sandoval-Ramirez JL, Mata-Marin JA, Huerta Garcia G, Gaytan-Martinez JE (2015). Responses to peginterferon alfa-2a vs alfa-2b plus ribavirin in a Mexican population with chronic hepatitis C. J Infect Dev Ctries.

[13] Pouresmaeeli M, Alavian SM, Keshvari M, Salimi S, Mehrnoush L (2015). Efficacy and Tolerability of Peginterferon alpha-2a and Peginterferon alpha-2b in Iranian Patients With Chronic Hepatitis C. Hepat Mon.

[14] Manns MP, McHutchison JG, Gordon SC, Rustgi VK, Shiffman M, Reindollar R (2001). Peginterferon alfa-2b plus ribavirin compared with interferon alfa-2b plus ribavirin for initial treatment of chronic hepatitis C: a randomised trial. Lancet.

[15] Neri S, Pulvirenti D, Bertino G (2006). Psychiatric symptoms induced by antiviral therapy in chronic hepatitis C: comparison between interferon-alpha-2a and interferon-alpha-2b. Clin Drug Investig.

[16] Fried MW, Shiffman ML, Reddy KR, Smith C, Marinos G, Goncales FL (2002). Peginterferon alfa-2a plus ribavirin for chronic hepatitis C virus infection. N Engl J Med.

[17] Olsson I, Mykletun A, Dahl AA (2005). The Hospital Anxiety and Depression Rating Scale: a cross-sectional study of psychometrics and case finding abilities in general practice. BMC Psychiatry.

[18] Bjelland I, Dahl AA, Haug TT, Neckelmann D (2002). The validity of the Hospital Anxiety and Depression Scale. An updated literature review. J Psychosom Res.

[19] Hung CI, Liu CY, Wang SJ, Yao YC, Yang CH (2012). The cut-off points of the Depression and Somatic Symptoms Scale and the Hospital Anxiety and Depression Scale in detecting non-full remission and a current major depressive episode. Int J Psychiatry Clin Pract.

[20] Chen CK, Tsai YC, Hsu HJ, Wu IW, Sun CY, Chou CC (2010). Depression and suicide risk in hemodialysis patients with chronic renal failure. Psychosomatics.

[21] Schaefer M, Capuron L, Friebe A, Diez-Quevedo C, Robaeys G, Neri S (2012). Hepatitis C infection, antiviral treatment and mental health: a European expert consensus statement. J Hepatol.

[22] Fabregas BC, Abreu MN, Dos Santos AK, Moura AS, Carmo RA, Teixeira AL (2014). Impulsiveness in chronic hepatitis C patients. Gen Hosp Psychiatry.

[23] Modabbernia A, Poustchi H, Malekzadeh R (2013). Neuropsychiatric and psychosocial issues of patients with hepatitis C infection: a selective literature review. Hepat Mon.

[24] Adinolfi LE, Nevola R, Lus G, Restivo L, Guerrera B, Romano C (2015). Chronic hepatitis C virus infection and neurological and psychiatric disorders: an overview. World J Gastroenterol.

[25] Bonkovsky HL, Woolley JM (1999). Reduction of health-related quality of life in chronic hepatitis C and improvement with interferon therapy. The Consensus Interferon Study Group. Hepatology.

[26] Thein HH, Maruff P, Krahn MD, Kaldor JM, Koorey DJ, Brew BJ (2007). Improved cognitive function as a consequence of hepatitis C virus treatment. HIV Med.

[27] Sarkar S, Jiang Z, Evon DM, Wahed AS, Hoofnagle JH (2012). Fatigue before, during and after antiviral therapy of chronic hepatitis C: results from the Virahep-C study. J Hepatol.

[28] Boscarino JA, Lu M, Moorman AC, Gordon SC, Rupp LB, Spradling PR (2015). Predictors of poor mental and physical health status among patients with chronic hepatitis C infection: the Chronic Hepatitis Cohort Study (CHeCS). Hepatology.

[29] Golden J, O'Dwyer AM, Conroy RM (2005). Depression and anxiety in patients with hepatitis C: prevalence, detection rates and risk factors. Gen Hosp Psychiatry.

[30] Martin-Santos R, Diez-Quevedo C, Castellvi P, Navines R, Miquel M, Masnou H (2008). De novo depression and anxiety disorders and influence on adherence during peginterferon-alpha-2a and ribavirin treatment in patients with hepatitis C. Aliment Pharmacol Ther.

